# Synthesis and Characterization of Mixed Iron-Manganese Oxide Nanoparticles and Their Application for Efficient Nickel Ion Removal from Aqueous Samples

**DOI:** 10.1155/2017/9476065

**Published:** 2017-07-19

**Authors:** Alessandro Buccolieri, Antonio Serra, Giuseppe Maruccio, Anna Grazia Monteduro, Sanosh Kunjalukkal Padmanabhan, Antonio Licciulli, Valentina Bonfrate, Luca Salvatore, Daniela Manno, Lucio Calcagnile, Gabriele Giancane

**Affiliations:** ^1^Department of Biological and Environmental Sciences and Technologies (DiSTeBA), Università del Salento, Via Per Arnesano, 73100 Lecce, Italy; ^2^Department of Mathematics and Physics “E. De Giorgi”, Università del Salento, Via Per Arnesano, 73100 Lecce, Italy; ^3^CNR NANOTEC-Istituto di Nanotecnologia, Via Per Arnesano, 73100 Lecce, Italy; ^4^IRCCS, National Institute of Gastroenterology “S. de Bellis”, Castellana Grotte, 70013 Bari, Italy; ^5^Department of Engineering for Innovation, Università del Salento, Via Monteroni, 73100 Lecce, Italy; ^6^Department of Cultural Heritage, Università del Salento, Via D. Birago, 73100 Lecce, Italy

## Abstract

Mixed iron-manganese oxide nanoparticles, synthesized by a simple procedure, were used to remove nickel ion from aqueous solutions. Nanostructures, prepared by using different weight percents of manganese, were characterized by transmission electron microscopy, selected area diffraction, X-ray diffraction, Raman spectroscopy, and vibrating sample magnetometry. Adsorption/desorption isotherm curves demonstrated that manganese inclusions enhance the specific surface area three times and the pores volume ten times. This feature was crucial to decontaminate both aqueous samples and food extracts from nickel ion. Efficient removal of Ni^2+^ was highlighted by the well-known dimethylglyoxime test and by ICP-MS analysis and the possibility of regenerating the nanostructure was obtained by a washing treatment in disodium ethylenediaminetetraacetate solution.

## 1. Introduction

About 20% of the world's population suffer from the so-called nickel allergic contact dermatitis [1, 2] and 30% of them are affected by the systemic nickel allergy syndrome (SNAS) [3, 4]. Such a disease is characterized by both cutaneous signs and extracutaneous symptoms such as gastrointestinal pains, chronic fatigue syndrome, and intense headache [5]. Furthermore, SNAS is a consequence of nickel accumulation in the patients and the allergic reaction can be promoted even by a very small amount of nickel taken during a relatively long period of time [6]. Therefore, SNAS patients need to follow a nickel-poor diet even though it is very difficult since nickel is ordinarily used in metal alloys (coins, watchstraps, and jewels) as well as in foodstuffs. In fact, nickel is widely used to form steel alloys and is also used as a catalyzer for chemical processes [7]; moreover, it is even used for particular dentistry applications and in nickel-cadmium batteries. Anthropogenic presence of nickel in the soil results in an increase of the Ni concentration in foods and in water, further reducing the foodstuffs suitable for the SNAS patients. For these reasons, a great deal of attention has been paid to developing methods for removing nickel ion from water, foods, juices, and vegetable extracts [8–10]. In particular, the use of nanoparticles to sequestrate and to remove heavy metal ions and pollutants is increasingly reported in the literature [11–14]. Nanostructures of absorbing inorganic materials, such as alumina [15], titanium oxides [16], cerium oxide [17], and zeolites [18], were employed to remove heavy metal ions from wastewater and they were synthesized in order to optimize the geometry of the porous absorbing nanostructures [19]. An important class of nanoparticles used to remove heavy metal ions from aqueous solutions is represented by the iron oxide nanoparticles. Such structures are particularly appealing since it is possible to easily remove the nanoparticles and the adsorbed pollutants by a simple application of an external static magnetic field. Naked [20] as well as capped [21, 22] iron oxide-based nanoparticles have been used to remove heavy metal ions from water and the mechanism has been studied as a function of pH, temperature, and contact time.

Here, we propose a very simple and inexpensive method to synthesize iron oxide nanoparticles, in particular a maghemite form of Fe_2_O_3_, with a tunable amount of Mn. The presence of manganese in the iron oxide-based nanostructures enhances the interaction with the Ni^2+^ dissolved in the aqueous solution, preserving the superparamagnetic characteristic of the maghemite nanoparticles [23]. The application of a simple neodymium magnet to the solution containing nickel ion and (*γ*-Fe_2_O_3_)-Mn nanostructures allows removing Ni^2+^ and decontaminating, in a few minutes, the samples without preliminary procedures.

## 2. Materials and Methods 

### 2.1. Synthesis of the (*γ*-Fe_2_O_3_)-Mn Nanostructures

A modification of the traditional method of coprecipitation to obtain maghemite and magnetite nanoparticles [24, 25] was employed to synthesize the Fe-Mn nanostructures [26].

All the reactions were carried out in Milli-Q grade water and all the compounds and solvents were purchased from Sigma-Aldrich and used without any further purification. Sodium hydroxide solution (0.5 M) was added dropwise to the FeCl_3_/MnCl_2_ solutions to reach a pH value of 11 and gently stirred. A dark precipitate was obtained just after the addition of sodium hydroxide and it was washed three times with ethanol and ultrapure water. Finally, the solutions were centrifuged and the solid phase of each sample was recovered by the use of a neodymium magnet. As reported in Table 1, the manganese weight percent (%) used in the synthesis and the effective presence of the Mn element quantified by EDS analysis are in reasonable agreement, with small changes in Mn12 and Mn50 samples.

pH value of the samples was modulated by means of the addition of hydrochloric acid and sodium hydroxide to the water solution.

### 2.2. Morphological and Structural Characterization

X-ray diffraction (XRD) data were collected using a Rigaku diffractometer (model Miniflex), employing Cu K*α* radiation at 30 kV and 100 mA operating in step-scan mode from 10° to 80°, with a 0.020° step and scan speed of 0.25°·min^−1^.

A transmission electron microscope (Hitachi, model 7700) was used to perform transmission electron microscopy (TEM) observations. Suitable samples for TEM observations were prepared by casting an ethanol suspension of nanoparticles onto a standard carbon coated copper grid [27]. The analysis of the nanostructures' morphology was carried out from bright field images. Information about the crystalline structures of the nanoparticles was obtained by small area electron diffraction (SAED).

N_2_ adsorption/desorption measurements at −196°C were performed with a BET NOVA 2000e (Quantachrome Instruments, USA) apparatus. The samples were degassed for 3 h at 105°C. Specific surface area (SSA) was determined by multipoint Brunauer–Emmett–Teller (BET) method using the adsorption data in the relative pressure range of 0.05–0.35. The pore size distribution and pore volume were calculated from the desorption isotherm using Barret–Joyner–Halenda (BJH) method.

### 2.3. Magnetic and Spectroscopic Characterization

The magnetic behavior of the nanostructures was investigated by using a vibrating sample magnetometer (VSM, Cryogenic Ltd.). The magnetization curves (M-H) were recorded at 300 K on dry samples packed in gelatin capsules over the field range comprised between −10 kOe and 10 kOe and were normalized by dividing by the magnetization at 10 kOe in order to allow an easier comparison.

Raman spectra were acquired with a Renishaw InVia Raman Microscope by using a 532 nm laser with a 100x objective. Laser power was set at 15 mW in order to prevent further oxidation of iron oxide. 50 scans were recorded for each sample.

UV-visible absorption spectra were assessed by means of a T80 UV-Vis Spectrometer (PG Instruments Ltd.).

### 2.4. Preparation of the Instant Tea Samples for the Nickel Ion Removal

Tea beverage used for nickel decontamination was prepared by dissolving 2 g of instant powdered tea in a nitric acid aqueous solution 1% (v/v). The solution was sonicated for 15 minutes and then filtered with a 0.45 *μ*m pore filter paper.

Nickel and iron concentrations were measured by inductively coupled plasma mass spectrometry (model iCAP Q, Thermo Scientific, Waltham, MA, USA) in static conditions. An aliquot portion (25 *μ*L) of each sample was diluted with ultrapure nitric acid 1% (v/v).

## 3. Results and Discussion

### 3.1. Morphological, Structural, and Spectroscopic Characterization of the Nanostructures

The stoichiometric Fe : Mn ratio strongly influences the physicochemical characteristics of the nanostructures and, in particular, their morphological and structural features.

The stoichiometry ratio between FeCl_3_ and MnCl_2_ was tuned in order to obtain nanostructures with different features. The samples were labelled as reported in Table 1.

**Figure 1:**
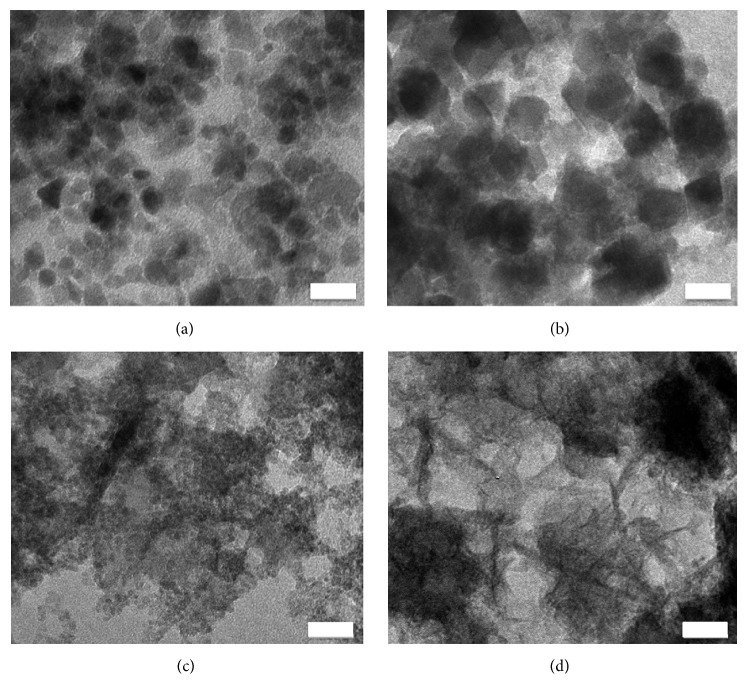
Morphology changes induced by the presence of Mn in the nanostructures' formation highlighted by transmission electron microscopy. The stoichiometry ratio Fe : Mn was (a) 100 : 0, (b) 88 : 12, (c) 75 : 25, and (d) 50 : 50. Scale bar = 20 nm.

In Figure 1, TEM images of the samples Mn0 and Mn12 (Figures 1(a) and 1(b), resp.) point out the dramatic variations induced by the different Mn percentages during the synthesis procedure. As shown in Figure 1(a), almost spherical nanostructures of about 10 nm were observed for the Mn0 sample, whereas the presence of manganese doping in the Mn12 sample induces the formation of nanostructures of about 20 nm to 40 nm.

TEM images of the samples Mn25 and Mn50 (Figures 1(c) and 1(d)) suggest that an excess of manganese inhibits the formation of the nanostructures and the quality of XRD spectra becomes strongly affected.

**Figure 2:**
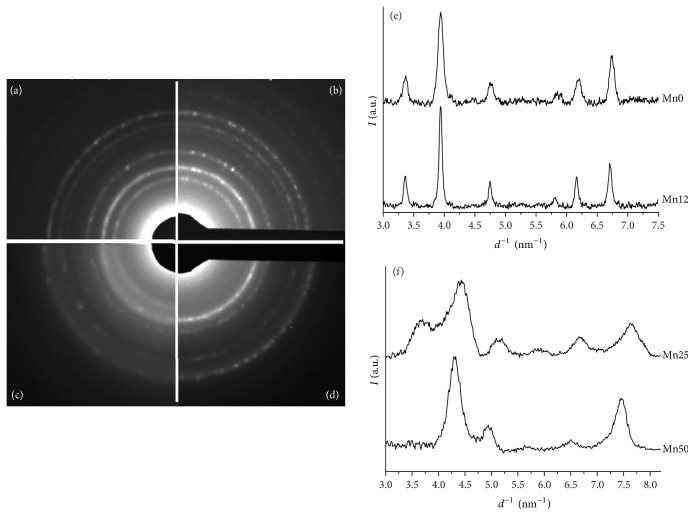
SAED measurements carried out on the samples: (a) Mn0, (b) Mn12, (c) Mn25, and (d) Mn50. (e) XRD spectra of Mn0 and Mn12 are plotted in the graph. Line profiles from SAED patterns of the samples Mn25 and Mn50 are reported in (f).

SAED measurements (Figure 2(a)) and XRD spectra (Figure 2(e)) suggest that Mn0 is in the *γ*-Fe_2_O_3_ (maghemite) phase. The presence of manganese in the sample Mn12 promotes the formation of larger and less regular structures even though the *γ* phase of Fe_2_O_3_ is preserved as highlighted by XRD and SAED measurements (Figures 2(e) and 2(b), resp.) with a slight increase of the unit cell (Mn_*x*_Fe_1−*x*_)_2_O_3_ from 0.833 nm to 0.838 nm.

SAED patterns, obtained for the samples Mn25 and Mn50 (Figure 2(f)), show crystallographic structures completely different from those observed for the samples Mn0 and Mn12.

SAED intensity profile, plotted as a function of *d*^−1^ (nm^−1^), of the sample Mn25 presents the typical peaks of both maghemite and jacobsite [28] and the presence of manganosite and pyrolusite influences the symmetry of the peaks at 4.9 nm^−1^ and 6.6 nm^−1^ [28].

When the manganese concentration is further increased (sample Mn50), SAED intensity profile evidences the formation of structures strongly oriented and characterized by typical features of the jacobsite [28].

**Figure 3:**
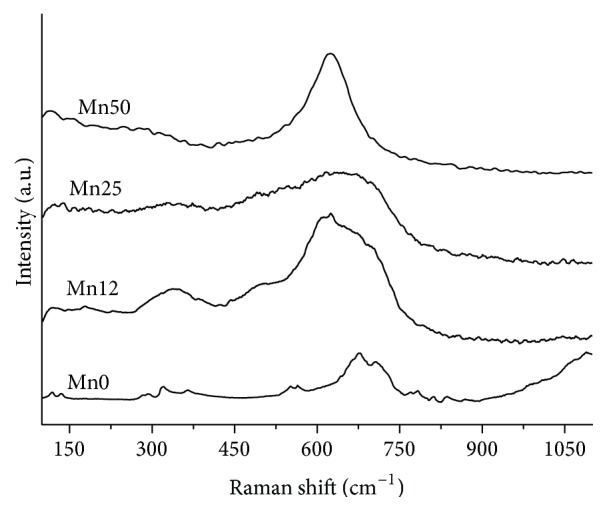
Raman spectra of the samples. Spectra were shifted along the intensity axis to better appreciate the difference in the Raman scattering profiles.

Confirmation of the proposed rationale for the SAED analysis was obtained by means of Raman spectroscopy. Raman spectrum of sample Mn0 (Figure 3) shows the typical absorption band of nanostructured maghemite [29] with three broad bands located at 320 cm^−1^ (T_2g_), 560 cm^−1^ (E_g_), and 680 cm^−1^ (A_1g_). The samples Mn12 and Mn25 (Figure 3) show the simultaneous presence of both maghemite vibrations and the intense MnO group stretching vibration of MnO_6_ with A_1g_ symmetry at 640 cm^−1^ [30]. Such a vibration is predominant in the spectrum of the sample Mn50 according to the strongly oriented jacobsite crystals evidenced by the SAED analysis.

### 3.2. Magnetic Characterization

**Figure 4:**
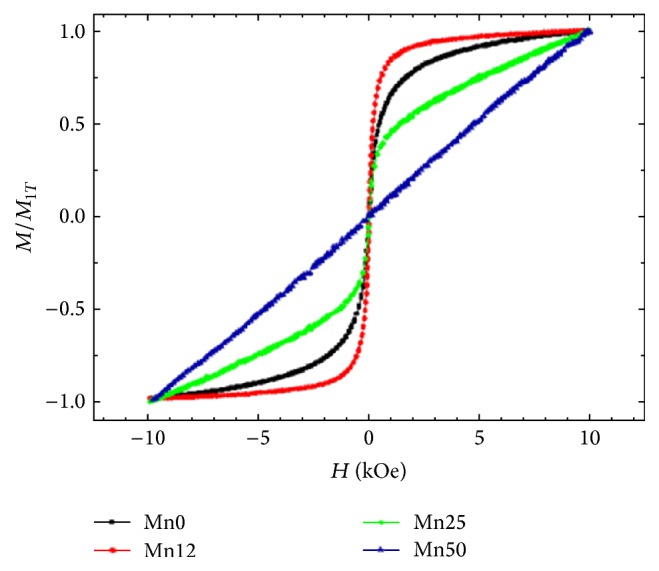
Normalized magnetization curve at the value of 1*T* of the (*γ*-Fe_2_O_3_)-Mn nanoparticles as a function of Mn concentration recorded at 300 K.

Figure 4 shows the normalized magnetization as a function of the applied magnetic field for the coprecipitated (*γ*-Fe_2_O_3_)-Mn nanoparticles in order to facilitate a comparison of their different response. The magnetization curves have been recorded at 300 K and normalized by dividing by the magnetization at 10 kOe. The magnetic behavior of the crystalline *γ*-Fe_2_O_3_ nanoparticles considerably changes by varying the Mn concentration from 12 to 50% (wt.%). In particular, the samples Mn0 and Mn12 exhibit a typical superparamagnetic behavior with no remanence and coercivity, which can be related to the small size of these magnetic nanoparticles. Among them, the sample Mn12, which preserves the same structure of the Mn0 sample (according to XRD results), achieves magnetization saturation at lower field strength and shows improved magnetic response with respect to the bare *γ*-Fe_2_O_3_ nanoparticles (sample Mn0) [31]. On the other hand, the sample Mn50 shows a linear magnetization trend [32], while sample Mn25 shows a magnetic response intermediate between samples Mn12 and Mn50, with a symmetrical sigmoidal shape of the magnetization curve, without reaching saturation magnetization. This suggests that the sample Mn25 is composed of both manganese oxides and mixed oxide phases. The observed changes from a superparamagnetic to an antiferromagnetic response as the Mn content increases (from sample Mn0 to sample Mn50) can be related to the structural variations evidenced by XRD and Raman characterizations.

### 3.3. Nickel Ion Removal from Lab-Prepared Aqueous Samples and Food Matrices

Fe-Mn nanostructures are able to remove heavy metal ions and organic pollutants as a consequence of the porous nanostructures [14, 33] and the modifications induced by manganese doping and the surface chemistry of the nanoparticles [23, 34].

The low magnetic response highlighted by the samples Mn25 and Mn50 prompted us to exclude them from the adsorption experiments. In fact, the quick removal of the Ni^2+^/iron oxide-based nanostructures from the food matrix represents a fundamental requisite for the industrial application of such technology.

**Figure 5:**
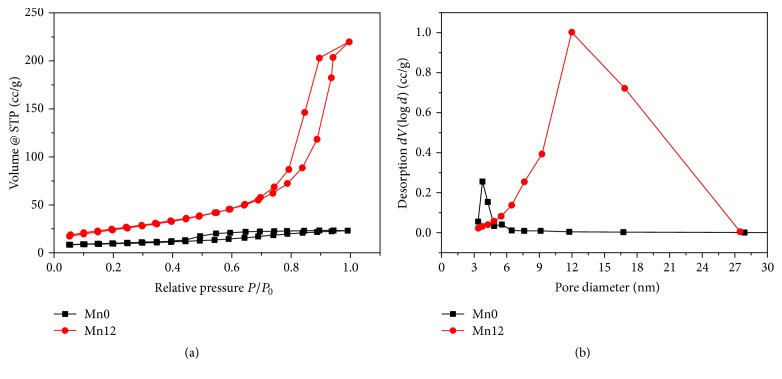
(a) N_2_ adsorption/desorption isotherms and (b) pore size distribution for Mn0 (black squares) and Mn12 (red points) samples.

In the present work, to verify the modification induced by the manganese to iron substitution in the nanoporosity of the nanoparticles, the N_2_ adsorption/desorption isotherms of Mn0 and Mn12 were analyzed at −196°C. As shown in Figure 5(a), for Mn12, the adsorption branch of the nitrogen isotherm is at a higher pressure range (0.7–0.9) with a higher BET specific surface area (SSA) and pore volume of 90 m^2^/g and 0.32 cc/g, respectively, whereas in case of Mn0 the adsorption branch of the nitrogen isotherm is shifted to a lower pressure range (0.45–0.8) and the BET surface area and the mesopore surface and volume decreased to 30 m^2^/g and 0.03 cc/g, respectively.

Mn12 displays the typical type IV adsorption isotherm. A hysteresis loop is also present corresponding to a typical mesoporous material [35].  Figure 5(b) shows the pore size distribution of both samples derived by BJH method on the desorption curve. Mn12 sample has an average pore diameter of 12 nm confirming the mesoporous structure whereas Fe_2_O_3_ has an average pore diameter of 3 nm corresponding to a microporous structure. The results of BET investigations look compliant with TEM analysis and confirm the great extent of microstructural changes induced by manganese substitution [23, 34].

The effective capability of the nanostructures to remove metal ions, and in particular nickel ion, from aqueous solutions was tested both on lab-prepared and on food samples.

**Figure 6:**
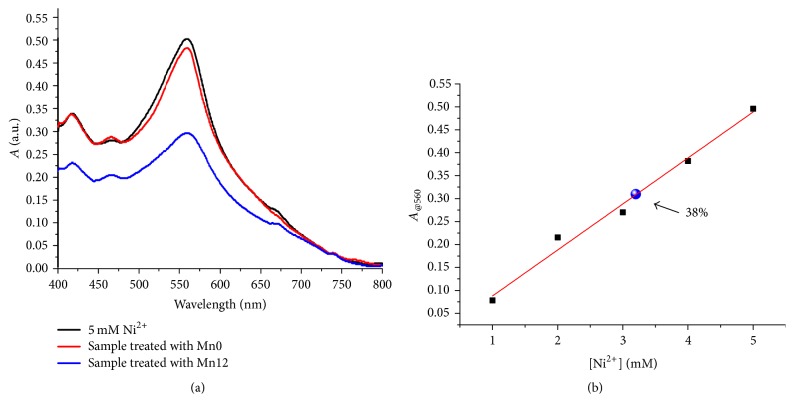
(a) Effect of the treatment carried out by Mn0 suspension (red line) and Mn12 suspension (blue line) evaluated by means of the dimethylglyoxime test. The black line represents the UV-visible spectrum of Ni^2+^/dimethylglyoxime complex at an ion concentration of 5 mM. (b) Absorbance intensity at 560 nm of the dimethylglyoxime-nickel complex as a function of nickel ion concentration. The blue circle represents the drastic decrease induced by the Mn12 (from a nickel concentration of 5 mM to 3.2 mM).

Nickel chloride was dissolved in ultrapure water in the concentration of 5 mM and the solutions were treated both with Mn0 and with Mn12 suspension in a concentration of 850 ppm and 722 ppm, respectively, evaluated by means of ICP-MS. The suspensions were gently stirred for 10 minutes in order to ensure good contact among the nanostructures and the heavy metal ion. A neodymium magnet was used to recover the magnetic nanostructures at the bottom of the flasks. The total content of nickel in the supernatant was evaluated by means of the well-known dimethylglyoxime test. As it is evident in Figure 6(a), the effect of Mn0 on total nickel concentration is almost negligible; on the contrary, the Mn12 nanostructures induce a reduction of Ni^2+^ of about 38% after 10 minutes of treatment only, reaching a value of 3.2 mM (blue circle in Figure 6(b)), confirming the crucial role of manganese in the nickel ion removal.

Starting from the percent of nickel removed by means of the presence of the Mn12 nanoparticles, it was possible to calculate that 1 mg of Mn12 can remove 0.16 mg of Ni^2+^. This value is lower than the state of the art of the nanoparticles' removal capability [15, 22], even though it is interesting to observe that the calculation of total nickel concentration was carried out only after 10 minutes of contact time with Mn12. In fact, as reported by Sharma and collaborators [15], a longer contact time ensures better adsorption and successive higher removal of the metal ions from the water solution.

The influence of pH on the ability of Mn12 to remove nickel ion from aqueous solutions was verified at four different pH values (3, 5, 7, and 8). At alkaline values, the nickel solubility is reduced and therefore these values were not considered [15]; for pH values lower than 2, the stability of iron oxide is affected. The dimethylglyoxime method was used to estimate the nickel ion amount after the treatment procedure with Mn12 at pH values of 3, 5, 7, and 8. The percent of Ni^2+^ removal was estimated to be 32.7%, 38%, 39%, and 39.2% for the solution at pH values of 3, 5, 7, and 8, respectively (each measurement was repeated on 3 different samples). It can be concluded that, in the investigated range, the efficiency of nickel ion removal increases as a function of pH, reaching an asymptotic value at about pH 7 [15].

**Figure 7:**
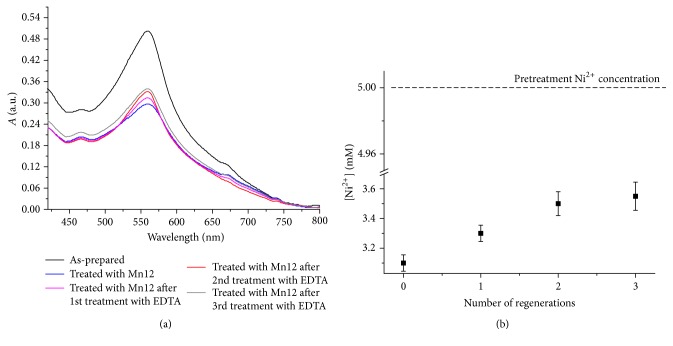
(a) Reusability of Mn12 nanostructure for Ni^2+^ decontamination after EDTA treatments revealed by dimethylglyoxime test and (b) influence of the number of regenerations on the Ni^2+^ removal.

The adduct Mn12/nickel ions were treated with a solution of 0.1 M disodium ethylenediaminetetraacetate dihydrate (Na_2_EDTA) for 3 hours in order to evaluate the possibility of reusing the nanoadducts to remove Ni^2+^. Na_2_EDTA is a well-known chelating agent for nickel ion that forms very stable complexes with the metal ions. After 3 hours, the magnetic nanostructures were removed by means of the application of a magnet and the efficacy of the Mn12 regenerated nanostructures for nickel removal was checked by the dimethylglyoxime test. As reported in Figures 7(a) and 7(b), the efficacy of the nanostructures for removing the nickel ion is still considerable after three application/regeneration procedures, even though a decrease of about 8% in the capability to trap the Ni^2+^ ions can be observed, probably as a consequence of the interaction among EDTA and Mn12 nanoparticles. It can be supposed that some binding sites on the surface of Mn12 nanoparticles are saturated by EDTA with an interaction strong enough to remain after the washing step. This phenomenon competes with the Mn12/Ni^2+^ adduct formation inducing a slight loss of the capability of Mn12 to remove nickel ion.

In order to exclude the possibility of contaminating the water solution with iron and manganese ions, the nanostructures' stability was tested. ICP-MS was used to evaluate the presence of iron and manganese ions. Ultrapure water solutions containing Mn25 at different pH values (3, 5, 7, and 12) were kept at 25 degrees for 20 days and an aliquot of samples' supernatant was withdrawn each day and measured by ICP-MS. In each sample, iron and manganese ions' concentration was under the ICP-MS limit of detection, even though it is worth observing that the paramagnetic behavior of Mn25 kept at basic pH was slightly affected, probably as a consequence of the formation of larger aggregates [36].

**Figure 8:**
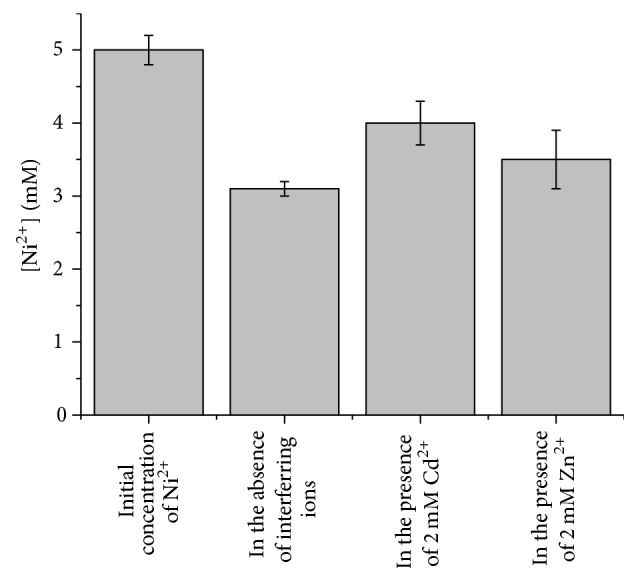
Effect of the presence of Cd^2+^ and Zn^2+^ on the ability of Mn12 to remove nickel ions from an ultrapure water solution containing 5 mM of Ni^2+^ at pH 7. Each measurement was repeated on 3 different samples.

It is known in the literature that similar nanostructures are able to remove different heavy metal ions [23]; therefore, the effect on the nickel removal ability of two interfering ions, Zn^2+^ and Cd^2+^, was checked. As reported in Figure 8, the presence of Cd^2+^ strongly reduced the nickel abatement in the presence of Mn12: competing adsorption of cadmium and nickel ions on the surface of the nanoparticles takes place, reducing the available sites for the absorption of Ni^2+^. A similar mechanism, despite being less evident than the case observed for cadmium ion, was recorded in the presence of Zn^2+^. These experimental lines of evidence suggest the Mn12 system as an absorbing material for the total content of heavy metal ions in wastewater or aqueous foodstuff.

Real samples of instant tea were prepared according to the procedure reported in the Materials and Methods, in order to check the efficacy of Mn12 even in complex matrices for the nickel capture. Mn12 concentration was estimated to be 25 mg/L. The experiment was carried out in order to evaluate the possibility of capturing and removing nickel ion from instant tea, because it could represent a very intriguing achievement. The nickel concentration, before treatment with Mn12 suspension, was measured to be 5.3 ± 0.1 ppm, according to the data reported in the literature [37]. After the treatment with Mn12, the nickel concentration was lower than the limit of detection of the technique. This result confirms that the iron-manganese oxide Mn12 nanoparticles are able to capture and drastically remove nickel ion from liquids and beverages, opening a wide range of practical applications.

## 4. Conclusions

Iron-manganese oxide nanoparticles were synthesized by a very simple and quick coprecipitation method starting from MnCl_2_ and FeCl_3_. Morphological and structural characterization, obtained by Raman spectroscopy, TEM, SAED, N_2_ adsorption/desorption, and XRD, revealed that the presence of manganese strongly influences the crystallinity and the porosity of the nanoparticles.

Magnetization curves were recorded for all samples and a superparamagnetic behavior of the nanostructures obtained using 100 : 0 (Mn0) and 88 : 12 (Mn12) Fe : Mn weight ratio was observed.

Adsorption/desorption curves revealed that Mn12's porosity is enhanced tenfold nearly when compared to the sample Mn0. This feature was used to remove nickel ion from lab-prepared samples and instant tea aqueous solution. Both dimethylglyoxime test and ICP-MS measurements confirmed that the porous structure of Mn12 promotes fast and complete nickel ion capturing. The magnetic Mn12/Ni^2+^ adduct was removed by the application of a neodymium magnet and treated by 0.1 M of Na_2_EDTA. This procedure allowed highlighting the possibility of regenerating the nanostructures to decontaminate water and food matrices several times.

## Figures and Tables

**Table 1:** Weight percent (wt.%) and effective concentration of manganese and Fe in the studied samples (the values were normalized considering that Fe (%) + Mn (%) = 100%).

Sample	MnCl_2_ (wt.%)	FeCl_3_ (wt.%)	Normalized Mn (%) in the nanostructure	Normalized Fe (%) in the nanostructure
Mn0	0	100	0	100
Mn12	12	88	16	84
Mn25	25	75	25	75
Mn50	50	50	60	40
